# Prevalence of perceived stigma and associated factors among primary caregivers of children and adolescents with mental illness, Addis Ababa, Ethiopia: Cross-sectional study

**DOI:** 10.1371/journal.pone.0261297

**Published:** 2021-12-20

**Authors:** Woredaw Minichil, Wondale Getinet, Tilahun Kassew

**Affiliations:** Department of Psychiatry, College of Medicine and Health Sciences, University of Gondar, Gondar, Ethiopia; Columbia University College of Physicians and Surgeons, UNITED STATES

## Abstract

**Background:**

Mental illness exposes persons to stigma and this stigma also affects family caregivers of persons with mental illness. The objective of the study was to assess the prevalence of perceived stigma and associated factors among primary caregivers of children and adolescents with mental illness, Addis Ababa, Ethiopia.

**Methods:**

A cross-sectional study design and systematic random sampling technique were used to recruit 408 participants at St. Paul’s Hospital Millennium Medical College and Yekatit-12 Hospital Medical College, Addis Ababa, Ethiopia. We collected the data by face-to-face interview. Devaluation of Consumer Families Scale was used to measure perceived stigma. Patient Health Questionnaire-9 and Oslo-3 social support scale were the instruments used to assess the factors. Coded variables were entered into Epidata V.3.1 and exported to SPSS V.21 for analysis. Binary logistic regression was used for analysis.

**Result:**

A total of 408 participants were interviewed, with a response rate of 96.5%. The magnitude of perceived stigma was 38.5% with 95% CI (33.6-43.1). Majority (68.6%) of the respondents were female. In the multivariate logistic regression, being mother (AOR = 2.8, 95% CI: 1.59, 4.91), absence of other caregiver (AOR = 2.0, 95% CI: 1.15, 3.49), poor social support (AOR = 3.9, 95% CI: 1.59, 6.13), and symptoms of depression (AOR = 2.9, 95% CI: 1.88, 3.65) were factors significantly associated with perceived stigma.

**Conclusion:**

The prevalence of perceived stigma among primary caregivers of children and adolescents with mental illness was high. Being mother, absence of other caregiver, poor social support, and symptoms of depression were factors significantly associated with perceived stigma.

## Introduction

Across the world, the burden of mental health problems is increasing [1]. Mental illness accounted for 13% of world disease burden and it will increase to 15% by the year 2020 [2,3]. Around 450 million people are affected by mental or behavioral disorders. Destructive effect of mental illness at personal and national level is highly significant [1,2,4,5]. In low- and middle-income countries, about 75% of people who need mental health service do not get any kind of intervention due to different reasons [6]. Stigma is a social process characterized by separation, rejection, and blame or discredit about an individual or groups [7]. Personal level stigma is the stigma that exists at the individual level [8]. High level of personal or self-stigma is also correlated with high psychological distress, decreased social functioning, and impaired quality of life [9]. The stigma of mental illness marks not only the patients but also their family caregivers [10]. In one of the earliest studies to evaluate stigma in caregivers and patients with schizophrenia, around half of the family members reported that they conceal the hospitalization of their patient [11]. A researcher suggests that the increased stress of caring for a child with attention-deficit/hyperactivity disorder plays a role in increased perceived stress, anxiety, and depression for caregivers [12]

Mental illness exposes persons to stigma and this stigma also affects family caregivers of persons with mental illness. Around 15% of the family caregivers feel that they are treated differently because of the mental illness of the person they care for. Eighteen percent of relatives of patients with schizophrenia or schizophrenia-like psychosis believed that they were stigmatized due to the mental illness of their relatives [13].

Perceived stigma (PS) is the perception or expectation of stigma which refers to people’s beliefs about attitudes of the general population toward their condition and themselves as being a member of potentially stigmatized group [14]. Families play an important role in the management of patients with mental illness. In developing countries, over 60% of patients with schizophrenia live with at least one primary caregiver [15]. Caregivers take care of their ill relatives at home; participate in treatment decision‑making and rehabilitation. Caregivers experience high levels of burden [16,17].

Psychosocial burden on family members of people with mental illness negatively affects both family members and the patients that they care for [18,19]. Stigma can lead to stress in family caregivers of people with mental illness [20]. Psychological disabilities of children have large social and economic burdens on the affected children and the societies at large [21]. Caregivers or families of stigmatized individuals may develop affiliate stigma and feel sad and helpless about their relation with the stigmatized individual [22]. Due to this, caregivers tend to conceal their status; withdraw from social relations, and isolate themselves from the patients in order to avoid association [23]. Many times, parents are blamed for mental health issues of their children, which creates a barrier in seeking help [24]. Perceived stigma predicts higher depression and social anxiety [25]. Family members of patients with mental illness experience shame, embarrassment, psychological suffering, and poor quality of life [26]. Family caregivers feel stigmatized due to negative stereotypes of mental illness of their children and thus either do not seek or delay seeking help [27,28].

On the other hand, healthcare professionals hold positive as well as negative stigmatizing perceptions of individuals with mental illnesses [29]. Stigma is one of the barriers that can prohibit individuals from getting appropriate treatment or care [30]. Previous studies have found that stigma-related beliefs among parents discourage help-seeking behaviors, and lessen the likelihood of appropriate mental health service use [10,31,32]. Stigma score was associated with unmet autism spectrum disorder care needs but not therapy hours or therapy types [33]. Different studies revealed that perceived stigma among caregivers of patients with mental illness was positively correlated with female sex, didn’t live with ill relative, presence of less severe positive symptoms in patients [11], female sex, being the only caregiver, long hours of spending in care [34], being the parents of the patient, giving care for female child, high educational status the patient, live in rural area, longer duration of illness, higher socio-economic status, higher degree of positive symptoms [35], absence of adequate social support, older age, depressive symptoms [36], and high score of affiliate stigma had significant correlation with the caregiver burden, depression, anxiety, self-esteem, quality of life and social support [37–39]. Although studies around the globe showed a high prevalence of perceived stigma, there is a dearth of evidence reported on the prevalence of perceived stigma especially among primary caregivers of children and adolescents with mental illness in Ethiopia.

### Objective

It was hypothesized that the prevalence of perceived stigma among primary caregivers of children and adolescents with mental illness is high; and different factors are associated with perceived stigma. Therefore, our study set out to assess the prevalence of perceived stigma and associated factors among primary caregivers of children and adolescents with mental illness in Addis Ababa, Ethiopia.

## Methods materials

### Study design and period

A cross-sectional study was conducted among primary caregivers of children and adolescents with mental illness attending outpatient department of child and adolescent psychiatry clinics from May to June, 2018.

### Study setting

The study was conducted at St. Paul’s Hospital Millennium Medical College (SPHMMC) and Yekatit-12 Hospital Medical College (Y12HMC) in Addis Ababa; the capital city of Ethiopia. These hospitals have been serving more than 5 million people in their catchment areas. Both hospitals are clinical and teaching hospitals, and providing organized child and adolescent psychiatric outpatient services. Child and adolescent psychiatric units have served for children and adolescents with mental illness for all working days. Daily, around 15-20 children and adolescents were given follow-up service at outpatient department of the child and adolescent psychiatry clinic in each hospitals.

### Participants and sampling procedure

Systematic random sampling technique was used to recruit 408 study participants. Clients with their caregivers were proportionally allocated into two hospitals (201 participants for SPHMMC, and 222 for Y12HMC) and determined the sampling interval “K = 2”, then the caregivers were interviewed every 2^nd^ in the clinic or outpatient department. Primary caregivers who aged 18 years and above, had been caring for the child on a regular basis, and those who gave informed consent were included in the study. Caregivers who had not been caring for the child but had been asked by the regular caregiver to accompany the child for appointment were excluded. Only the primary diagnosis of children that mainly bother caregivers was considered.

### Primary caregiver

In this study, we defined primary caregiver as an individual who aged 18 year and above, and provided care for at least 50% of total duration of the illness and made the decision with the clinicians about the treatment modality for a child with mental illness in the family [40].

In this study “"family history of mental disorder" means “presence of past or current history of mental disorder in the vertical/not siblings or horizontal/ relationship of caregivers either in father/grandfather or mother/grandmother.

### Sample size determination

The sample size was determined by using the single population proportion formula with the following assumptions: 50% prevalence of perceived stigma (because no published literature in Ethiopia especially the area and the topic of this study conducted), 5% margin of error (d), 95% confidence interval of certainty (alpha = 0.05), and 10% non-response rate. Based on these assumptions, the total sample was 423.

### Study variables

Perceived stigma among caregivers of children with mental illness was the dependent variable. Explanatory variables included socio-demographic factors like age, sex, religion, ethnicity, income, employment status, marital status, number of children, educational status, duration of care-giving, social support and caregiver’s relation to the child with mental illness; clinical factors including depression and chronic medical illness, and children related factors such as age, sex, duration of illness and types of mental illness.

### Data collection instruments

Data were collected by face-to-face interview using a semi-structured questionnaire by two psychiatric nurses using Amharic version of the questionnaire for a month. Data regarding children and adolescents were collected from secondary data or “clients’ file registration” and their caregivers. The questionnaire was designed in English and translated to Amharic and back to English to maintain its consistency. Training was given for data collectors on how to interview participants and clarified unclear questions. Furthermore, they were been aware about confidentiality and data management.

Perceived stigma was measured by Devaluation of Consumers Family Scale (DCFS). DCFS is a standardized interviewer administered scale comprising of 7 items regarding stigma that affect caregivers/family members. A total score (ranged from 7 to 28) was obtained by summing the scores from each of the 7 items. It had a 4 point Likert response options ranging from 1 “strongly disagree” to 4 “strongly agree”. We adopted this instrument from a validation study conducted on the extent to which caregivers believe most people devalue consumers and their families [41]. It showed a high internal consistency, reliability and a strong correlation with PS. We carried out a reliability analysis for the DCFS items and that it had high score (Cronbach’s α = 0.88). Based on this, greater or equal to the mean of the total score of the instrument was taken to declare perceived stigma in the current study.

Symptoms of depression were measured by Patient Health Questionnaire (PHQ-9). A total score of PHQ-9 ranges from 0 to 27. Each of the 9 items was scored from 0 “not at all” to 3 “nearly every day”. Its total sum score indicates that 0-4 “minimal”, 5-9 “mild”, 10-14 “moderate”, 15-19 “moderately severe” and 20-27 “severe” depression [42]. PHQ-9 has been validated in Ethiopian health care context and its specificity and sensitivity was 67% and 86% respectively. We have used a cut-off-10 and above because our concern was to show symptom severity suggestive of moderate to severe depression among the respondents [43].

Social support was assessed using the Oslo-3 social support scale. The internal consistency of OSS-3 was low with a Cronbach’s alpha coefficient of 0.50. The concurrent validity of OSS-3 with the depression sub-scale of Hospital Anxiety Depression Scale (HADS) was low but significant and inversely related. Its sum score ranges from 3 to 14, which has three broad categories: 3-8 “poor social support”, 9-11 “moderate social support”, and 12-14 “strong social support” [44].

### Data processing, analysis and result presentation

The data were coded and entered into Epi-data version 3.1, and exported to SPSS- 21 version for analysis. Descriptive statistics like frequency, percentage, mean and standard deviation were employed to describe the results of the data using table and figure. Bivariate logistic regression analysis was used to evaluate the associations between outcome and predictor variables. To control the confounding effects, variables with a p-value of < 0.2 in the bivariate analysis were fitted to the multivariable logistic regression model. Odds ratio with 95% confidence interval was used to interpret the associations between the explanatory and the dependent variables. Variables with a P-value of < 0.05 in the multivariate logistic regression were considered as statistically significant.

### Ethical consideration

Ethical clearance was obtained from the Ethical Review Board of St. Amanuel Mental Specialized Hospital and University of Gondar. Letter of permission was also obtained from SPHMMC. Written informed consent was obtained from all caregivers who participated in the study. Each respondent was informed about the risk, benefit, the time taken to interview and the objective of the study that might contribute to policymakers and other concerned bodies by giving valuable. Anybody who was not interested to participate in the study was not forced. Participants were informed that confidentiality was maintained throughout the study period. Participants with severe depression and suicidal behavior were referred to the nearby adult psychiatric clinic for further evaluation by the working clinician during the data collection period.

## Result

A total of 408 participants were enrolled in the study with a response rate of 96.5%. The mean age of the respondents was 42.95 ± 7.07(SD) years ranging from 22 to 60 years. Majority of the participants, 280 (68.6%), were female; 205 (50.2%), were Amhara in ethnicity. Two hundred fifty-three (62%) of the participants were married; 224 (54.9%) were employed either governmental or private companies. Two hundred sixty-one (64.0%) of the respondents were orthodox Christian; 321 (78.7%) live in the urban area. A significant number, 341 (83.6%), of the respondents had attended at least primary education. More than half, (54.7%), of the participants were mothers; the mean duration of care the caregivers gave was 4.22 + 3.43 (SD); 211 (51.7%) had other caregivers within the family. In this study, we employed 18 independent factors, of these; only four factors (being mother, absence of other caregiver, poor social support and depression) were significantly associated with perceived stigma in the multivariate logistic analysis (**Table 1**).

**Table 1:** Socio-demographic characteristics of caregivers of children and adolescents with mental illness at St. Paul’s Hospital Millennium Medical College and Yekatit-12 Hospital Medical College, Addis Ababa, Ethiopia, 2018 (n = 408).

Variables	Category	Frequency (n)	Percentage (%)
Age (in year)	Mean (42.95) ± SD (7.07)		
Duration of care (in year)	Mean (4.22) ± SD (3.43)		
Sex	Male	128	31.4
	Female	280	68.6
Ethnicity	Amhara	205	50.2
	Oromo	99	24.3
	Tigre	41	10.1
	Guragie	63	15.4
Marital status	Single	70	17.2
	Married	253	62.0
	Divorce	44	10.8
	Widow/er	41	10.0
Occupation	Governmental employee	135	33.1
	NGO employee	89	21.8
	Merchant	96	23.5
	Farmer	13	3.2
	House wife	75	18.4
Residence	Urban	321	78.7
	Rural	87	21.3
Education	No formal education	67	16.4
	Primary	117	28.7
	Secondary	90	22.1
	Tertiary	134	32.8
Relationship	Mother	223	54.7
	Father	145	35.5
	Sibling	40	9.8
Other caregiver	Absent	197	48.3
	Present	211	51.7

### Psychosocial and clinical factors of caregivers

Of the total participants, 78 (19.1%) had chronic medical illness (e.g., diabetes mellitus, hypertension); 57 (14%) history of mental illness in the family; 233 (57.1%) poor social support, and more than half or 235 (57.6%) depressive symptoms (**Table 2**).

**Table 2:** Clinical characteristic distributions of caregivers of children and adolescents with mental illness at child and adolescent psychiatric clinics of St. Paul’s Hospital Millennium Medical College and Yekatit-12 Hospital Medical College, Addis Ababa, Ethiopia, 2018 (n = 408).

Variables	Category	Frequency (n)	Percentage (%)
Chronic medical illness	No	330	80.9
	Yes	78	19.1
Family history of mental illness	No	351	86.0
	Yes	57	14.0
Social support	Poor	233	57.1
	Moderate	127	31.1
	Strong	48	11.8
Depressive symptoms	No	173	42.4
	Yes	235	57.6

### Children and adolescents related factors

The mean age of the children and adolescents with mental illness was 10.95 ± 3.37(SD) ranged from 2 to 17 years, and 209 (51.2%) were male. Almost half (49.5%) of the children and adolescents with mental illness were under the age of 10 years, and among children and adolescents with mental illness who were under the care of their family, 209 (51.2%) were diagnosed with Autism Spectrum Disorder/ASD and the mean of duration of illness was 9.54 ± 5.84 (SD) (**Table 3**).

**Table 3:** Characteristics distribution of children and adolescents with mental illness attending child and adolescent psychiatric clinics at St. Paul’s Hospital Millennium Medical College and Yekatit-12 Hospital Medical College, Addis Ababa, Ethiopia, 2018. (n = 408).

Variables	Category	Frequency (n)	Percentage (%)
Age (in year)	Mean(10.95) ± SD(3.37)		
Duration of illness (in year)	Mean(9.54) ± SD(5.84)		
Sex	Male	209	51.2
	Female	199	48.8
Diagnosis	Intellectual disability	76	18.6
	Autism spectrum disorder	209	51.2
	Attention-deficit/hyperactivity disorder	107	26.2
	Others	16	3.9

### Prevalence of perceived stigma

The mean of perceived stigma score among primary caregivers of children and adolescents with mental illness was 18.84 ± 4.83(SD). One hundred fifty seven (38.5%) of the respondents scored above the mean i.e., the prevalence of perceived stigma among primary caregivers of children and adolescents with mental illness was 38.5%. Among the respondents with perceived stigma, 150 (66%) were female by sex; 96 (61.1%) were married; 65 (28.6%) attended college and above; 75 (33%) were unemployed; 74 (47.1%) were mothers of children and adolescents with mental illness, and 107 (68.2%) had depressive symptoms. Ninety-six (61.1%) of the respondents with perceived stigma had no other caregiver at home, 83 (52.9%) had children and adolescents diagnosed with Autism Spectrum Disorder (ASD), and 115 (73.2%) had poor social support. Regarding the proportion of perceived stigma toward each item, 36.8% of the caregivers strongly agreed with the item “most people look down on families that have a member who is mentally ill living with them”, followed by ‘‘most people in my community would rather not be friends with families that have a relative who is mentally ill living with them” 34.1%. The least strongly agreed item was “most people believe their friends would not visit them as often if a member of their family were hospitalized for a serious mental illness” 9.3%. Items leveled as a1, a2 and a3 are reversed (**Table 4**).

**Table 4:** Proportion of perceived stigma response of caregivers to each item at St. Paul’s Hospital Millennium Medical College and Yekatit-12 Hospital Medical College, Addis Ababa, Ethiopia, 2018 (n = 408).

Items	Negative responses		Positive responses	
	Strongly disagree (%)	Disagree (%)	Agree (%)	Strongly agree (%)
Most people in my community would rather not be friends with families that have a relative who is mentally ill living with them	19 (4.7)	121 (29.7)	129 (31.6)	139 (34.1)
Most people believe that parents of children with a mental illness are not just as responsible and caring as other parents^a1^	114 (27.9)	135 (33.1)	116 (28.4)	43 (10.5)
Most people look down on families that have a member who is mentally ill living with them	31 (7.6)	72 (17.6)	155 (38.0)	150 (36.8)
Most people believe their friends would not visit them as often if a member of their family were hospitalized for a serious mental illness	57 (14.0)	164 (40.2)	149 (36.5)	38 (9.3)
Most people do not treat families with a member who is mentally ill in the same way they treat other families^a2^	56 (13.7)	86 (21.1)	181 (44.4)	85 (20.8)
Most people blame parents for the mental illness of their children^a3^	39 (9.6)	92 (22.5)	169 (41.4)	108 (26.5)
Most people would rather not visit families that have a member who is mentally ill	41 (10.0)	124 (30.4)	180 (44.1)	63 (15.4)

### Factors associated with perceived stigma

In order to determine the association of explanatory variables with perceive stigma, bivariate and multivariate binary logistic regression analyses were carried out. In the bivariate analysis, characteristics of the primary caregivers like marital status, occupation, relationship with the children and adolescents, absence of other caregiver, family history of mental illness, sex of children/adolescents, type of diagnosis, perceived social support and depressive symptoms were found to be significantly associated with perceived stigma at a P-value < 0.2. These factors were fitted to multivariate logistic regression model for further analysis to control confounding effects. In the current study, we employed 18 independent factors, of these; only four factors (being mother, absence of other caregiver, poor social support and depression) were significantly associated with perceived stigma in the multivariate logistic analysis.

The odds of reporting perceived stigma was about 3 times higher among mothers compared to siblings (AOR = 2.82, 95% CI: 1.59, 4.91). Respondents who had no other caregiver were 2 times more likely to report perceived stigma compared to those who had other caregiver (AOR = 2.00, 95% CI: 1.15, 3.49). The likelihood of reporting perceived stigma was nearly 4 times higher among primary caregivers who had poor social support compared to those who had strong social support (AOR = 3.85, 95% CI: 1.59, 6.13). Moreover, the odds of reporting perceived stigma was approximately 3 times higher among participants who had symptoms of depression compared with those who had not such illness (AOR = 2.92, 95% CI: 1.88, 3.65) (**Table 5**).

**Table 5:** Bivariate and multivariate logistic regression analysis of factors associated with perceived stigma among primary caregivers of children and adolescents with mental illness attending at St. Paul’s Hospital Millennium Medical College and Yekatit-12 Hospital Medical College, Addis Ababa, Ethiopia, 2018. (n = 408).

Variables	Category	Perceived stigma		COR with 95 CI	AOR with 95 CI
		High	Low		
Marital status	Single	26	44	1.00	1.00
	Married	96	157	0.97 (0.56,1.67)	0.61 (0.32,1.15)
	Divorce	18	26	1.13 (0.59,2.17)	0.80 (0.38,1.70)
	Widow/er	17	24	1.16 (0.59,2.27)	0.92 (0.43,1.97)
Occupation	Government employee	50	85	1.00	1.00
	Private employee	35	54	1.10 (0.64,1.91)	0.91 (0.50,1.65)
	Merchant	40	56	1.21 (0.71,2.07)	1.11 (0.62, 2.01)
	Farmer	8	5	2.72 (0.84,8.77)	1.72 (0.48,6.15)
	House wife	24	51	0.80 (0.44,1.46)	0.81 (0.42,1.56)
Relationship	Mother	74	149	2.64 (0.37,1.11)	2.82(1.59, 4.91)*
	Father	52	62	1.08 (0.60,1.97)	0.95 (0.49,1.86)
	Sibling	31	40	1.00	1.00
Other caregiver	Absent	96	127	1.54 (1.03,2.30)	2.00(1.15, 3.49)*
	Present	61	124	1.00	1.00
Family history of mental illness	No	141	210	1.00	1.00
	Yes	16	41	0.58 (.31,1.08)	0.60 (0.31,1.18)
Child sex	Male	67	142	1.00	1.00
	Female	90	109	1.75 (0.17,2.62)	2.11 (0.82, 3.26)
Diagnosis	ID	24	52	1.00	1.00
	ASD	83	126	1.43 (0.82, 2.49)	0.80 (0.42,1.50)
	ADHD	44	63	1.51 (0.82,2.81)	1.17 (0.58,2.34)
	Others	6	10	1.30 (0.42,3.99)	1.16 (0.36,3.75)
Social support	Poor	115	118	4.87 (2.19,10.86)	3.85(1.59, 6.13)*
	Moderate	34	93	1.83 (0.78, 4.30)	1.40 (0.57,3.44)
	Strong	8	40	1.00	1.00
Depressive symptoms	No	50	123	1.00	1.00
	Yes	107	128	2.06 (1.36,3.12)	2.92(1.88, 3.65)*

## Discussion

The presence of stigma among children with emotional and mental/behavioral disorders has been supported by previous study finding [10]. Experience of stigma is not only limited to the clients but it can be also experienced by close relatives. However, this study is the first to examine perceived stigma among caregivers of children and adolescents with mental illness in Addis Ababa, Ethiopia.

Since the prevalence of perceived stigma among caregivers of patients with mental/behavioral disorders is now increasing worldwide, it needs a common understanding of its prevalence, impact and potential factors. Parents of individuals with mental or behavioral illness are commonly accused of causing the illness, and even the other family members are blamed for not caring patients with mental illness well [45]. Due to the above problem, caregivers/family members may develop poor health outcomes as the consequence of perceived stigma. Perceived stigma is correlated to the poor social interaction and inappropriate coping strategies [46]. Thus, monitoring the perceived stigma of the family members is a critical issue [47].

In this study, the prevalence of perceived stigma among primary caregivers of children and adolescents with mental illness was 38.5% with 95% CI (33.6, 43.4), which was in line with the studies conducted in the USA (41.3%), Zambia (39%) [48,49]. However, the finding in this study was higher than the prevalence reported in India (21%) [50]. The difference for this variation might be sample size, methodological difference and study population i.e., smaller sample size, mixed method and caregivers of patients with schizophrenia in India. On the other hand, the prevalence of the current study finding was lower than the studies conducted in the other part of Ethiopia (75%), Morocco (86.7%), Virginia (78%), Belgium (86%) [51–54] and China (65%) [36].

Concerning the factors associated with perceived stigma in the current study, caregivers who had poor social support were five times more likely to have perceived stigma compared to those with strong social support. The assumptions might be the reason that children and adolescents with mental illness living in the family are socially isolated, and are assumed as incompetent, unaccountable and irresponsible by the community. This association is similar with other studies conducted in another area of Ethiopia, Virginia, and China [51,55–57]. Caregivers who had no other persons helping them in the care of their child/adolescent with mental illness were at high risk of perceived stigma. Primary caregivers who had depressive symptoms were more likely to be exposed to perceived stigma than those who had not depressed. This could be the reason that caregivers with depressive symptoms are pessimistic and have feeling of discrimination by others. Another reason might be the fact that being depressed might contribute to frequent feeling of stigma. This is in agreement with a study conducted in different areas [57–59]. Being mother of children and adolescents with mental illness was significantly associated with perceived stigma. The possible reasons might be that mothers are easily affected by shame, ignorance, feeling of isolation from social relationships and guilty feeling because their neighbours usually blame them that they are the responsible individuals for the mental illness of their children.

### Strengths

Devaluation of Consumer Families Scale (DCFS), was used to measure perceived stigma, can serve as a reference in subsequent studies since it has good internal consistency. Relatively large sample size and probability sampling technique can make the study representative.

### Limitations

The interview was face to face, so that the study might be affected by social desirability bias. The nature of a cross-sectional study design might be only partially accounted for durable temporal relationships. Presence of comorbid illnesses or two/more diagnosis among children and adolescents were not identified; this may vary the burden of the caregivers.

## Conclusion

The prevalence of perceived stigma was high compared to the general population and most related study findings. Being mother, absence of another caregiver, poor social support and depression were predictors significantly associated with perceived stigma. Stigma can be addressed through different strategies. Therefore, we recommended protesting/ organized public demonstration, education/advocacy and awareness creation, health promotion and contact/communication and organizing anti-stigma campaigns because it helps to reduce negative consequences of mental illness stigma.
